# The Voluntary Hospitals Committee

**Published:** 1921-06-18

**Authors:** 


The Hospital

The Workers' Newspaper of Administrative Medicine and Institutional Life. Administration. National Insurance and Health.

No. 1828, Vol. LXX. SATURDAY, JUNE 18, 1921. PRICE TWOPENCE

### THE VOLUNTARY HOSPITALS COMMITTEE.

The issue in full of the final report of Viscount Cave's Committee enables one to examine the evidence and reasoning which led to the series of recommendations which were published in advance of the report. It is noticed from one of the appendices that the Committee heard in all ninety-three witnesses, who represented organisations having to do directly or indirectly with the question at issue. The Committee was therefore in a position to take a survey of the entire position of the voluntary hospitals of this country, which had not been possible for any body since the commencement of what is called "the hospital crisis."

The note of the report is best exemplified by the following quotation: "The voluntary hospital system, which is peculiar to the English-speaking peoples, is part of the heritage of our generation; and it would be lamentable if, by our apathy or folly, it is allowed to fall into ruin." We should like to say at once that a very considerable service has been rendered to the voluntary system by Viscount Cave and his colleagues, and that their painstaking work will do much to strengthen the system, apart from any aid which may ensue from the recommendations.

It is reassuring to discover that the financial position of the voluntary hospitals is not so grave as was usually thought, in fact during last year the deficits of the seventy-three hospitals of London whose income did not reach the expenditure amounted together to £463,413, while forty of the hospitals actually had an aggregate surplus of £97,240. The story as regards the Provinces is similar. Of 452 hospitals surveyed, 248 had deficits amounting in all to £501,282, while 204 had surpluses amounting to £220,126. In Scotland the aggregate surpluses were £169,865 and the deficits £73,623. In none of the instances quoted has allowance been made for sums allocated from the £200,000 granted by the National Relief Fund, nor with reference to London is the emergency distribution made by King Edward's Hospital Fund of £254,150 taken into consideration. We therefore have a fair opportunity of estimating the probable deficit for the coming year, which will probably be not far short of that for 1920—namely, £1,038,318—those fortunate hospitals which together have the surplus of £427,231 being probably able to look after themselves for the present. It is presumably on the basis of these figures that the Hospitals Committee have arrived at the proposed grant from the Exchequer of one million and, having in mind the well-known fact that the majority of hospitals have large schedules of outstanding repairs waiting for better times, have made the suggestion that a further half million should be provided for extensions and improvements within two years from date.

In answering the question, Is the Voluntary Hospital system worth saving? Lord Cave's Committee reply in terms which have been used by hospital experts for many years, but the expression of opinion has the advantage of coming from an entirely unprejudiced source and as the result of independent investigation. If the system falls to the ground, hospitals must be provided by the public, and the expense would be enormous. The three million given a year in voluntary subscriptions and donations and presumably the one million from endowments would cease, while, on the other hand, wages and other disbursements would rise and the payment of full remuneration to the medical and administrative staffs would at once become an urgent question. But the money lost to the State would be a small matter compared to the injury done to the welfare of the sick for whom the hospitals are provided, to the training of the medical profession, and to the progress of medical research. Upon these points much is said in the report, and a convincing picture of the difference between English and American hospitals when compared with those of some other countries is used to emphasise the argument (see also p. 190).

